# Factors Associated with Postpartum Depression among Women in Eastern Sudan: A Cross-Sectional Study

**DOI:** 10.3390/medicina60071167

**Published:** 2024-07-19

**Authors:** Khalid Nasralla, Saeed Omar, Ghusun Alharbi, Fai Aljarallah, Nadiah AlHabardi, Ishag Adam

**Affiliations:** 1 Department of Obstetrics and Gynecology, College of Medicine, Qassim University, Buraidah 51452, Saudi Arabia; k.hashim@qu.edu.sa (K.N.); ghousonsuliman@gmail.com (G.A.); faisaleh8741@gmail.com (F.A.); ia.ahmed@qu.edu.sa (I.A.); 2 Faculty of Medicine, Gadarif University, Gadarif 32211, Sudan; drsaeedomar@yahoo.com

**Keywords:** postpartum, depression, pregnancy, age, parity

## Abstract

*Background and Objectives*: Postpartum depression is one of the most common complications of childbirth. While the epidemiology of postpartum depression has been extensively studied in African countries, there is little published data on the topic in Sudan. In addition, no studies have been conducted in Eastern Sudan. This study aims to evaluate the factors associated with postpartum depression among Sudanese women in Gadarif in Eastern Sudan. *Materials and Methods*: A cross-sectional study (using the systematic random sampling technique) of women presenting to Gadarif Maternity Hospital for postnatal follow-up within six weeks of childbirth was conducted. A questionnaire was used to collect sociodemographic information, and the Edinburgh Postnatal Depression Scale was used to assess postpartum depression. *Results*: Three hundred women were enrolled in the study. The median (interquartile) age and parity were 30.0 (25.0–34.0) years and 2 (1–4). Thirty-one (10.3%) of the women had postpartum depression. A univariate analysis showed that a past history of depression was the only factor associated with postpartum depression (OR = 3.04, 95% CI = 1.03–8.97). Other investigated factors (age, parity, educational level, occupation, history of previous miscarriage or intrauterine fetal death, a family history of depression, financial support, medical insurance, whether the pregnancy was planned or not, and if the gender of the newborn was known before delivery) were not associated with postpartum depression. *Conclusions*: The current study showed that 1 out of 10 women had postpartum depression that was associated with a past history of depression. Factors that have been reported to be associated with postpartum depression in African countries (age, parity, education, and occupation) were not found to be associated in this study. Mental health assessment needs to be employed for women in their antenatal and postpartum periods.

## 1. Introduction

Depression is a worldwide public health problem, and women, in particular, are at a higher risk of experiencing mental disorders such as stress, depression, and psychosis during pregnancy and the postpartum period [1,2]. Postpartum depression is one of the most common complications of childbirth and may pass unnoticed if not properly assessed [3]. Although depressive episodes are most common in the first six weeks, women remain susceptible up to 14 months after delivery [4]. Postpartum depression is associated with stigma [5], a wide range of emotional, physical, and behavioral changes that can affect the mother, her newborn baby, and the whole family [6]. The major symptoms include loss of interest, sleeping difficulties, fatigability [6], lack of concentration, agitation, appetite changes, and suicidal thoughts [7]. Moreover, postpartum depression can affect the bond between the mother and her newborn, which may consequently affect the child’s growth, nutrition, and future mental health, and may lead to infanticide [6].

There is great variation in the prevalence of postpartum depression, which ranges between 14.8% in developed countries and 19.99% in countries with fewer resources [2]. Moreover, different rates of prevalence have been reported in different countries in Africa [8,9,10,11,12] for example, the prevalence of postpartum depression in Ethiopia (12.2–33.8%) [9], Kenya (27.1%) [13], Uganda (27.1%) [10], Nigeria (37.8%) [4], Somalia (59.9%) [14], Cameroon (31.8%) [15], Burkina Faso (44.0%) [6], South Africa (39.96%) [2], and Tanzania (12.2%).

Factors such as low socioeconomic status [8], a past history of depression [7], a family history of mental disorders [16], stressful life events [2,8], unplanned pregnancy [17], age, parity [18], loss of familial support [8], domestic violence [9], educational level [19], gender of the baby [8], previous infant loss [7], occupation [20], a history of miscarriages [21], and adverse pregnancy outcome [22] are the reported risk factors for postpartum depression. While the epidemiology (prevalence and associated factors) of postpartum depression has been extensively studied in African countries [8,9,10,11,12,13,14,23,24], there is little published data on the topic in Sudan [17,19], and no studies have been conducted in Eastern Sudan. Sudan is the third largest country in Africa, and a high prevalence of maternal morbidity and mortality has been reported [25]. Generally, the health system (including maternal and mental health) is fragile in Sudan, with low health indicators, especially in the eastern part of Sudan [26].

This study aims to evaluate the associated factors of postpartum depression among Sudanese women in Gadarif Maternity Hospital. The hope is to fill the knowledge gap regarding the magnitude and risk factors of postpartum depression among Sudanese women to help healthcare providers and decision-makers construct evidence-based policies and improve mental health screening services at the level of primary healthcare units for early detection and early intervention.

## 2. Materials and Methods

### 2.1. Study Area

Gadarif Maternity Hospital is located in the city of Gadarif and is the largest government-run maternity hospital in Eastern Sudan. It provides services to all women in the state of Gadarif. The hospital staff covers the antenatal and postnatal clinics free of charge.

### 2.2. Subjects and Study Design

A cross-sectional study of women presenting to Gadarif Maternity Hospital for postnatal follow-up within six weeks of childbirth was conducted. The guidelines of the Strengthening the Reporting of Observational Studies in Epidemiology (STROBE) initiative were strictly followed [27]. The study was conducted from February 2022 to August 2022.

### 2.3. Inclusion and Exclusion Criteria

Sudanese women aged ≥ 18 who had given birth within the previous six weeks and agreed to participate were enrolled in the study. Women who refused to participate or could not communicate due to a language barrier were excluded.

### 2.4. Sampling Technique

The required sample of 300 women was enrolled using the systematic random sampling technique. According to hospital records, 877 women visited the postnatal care unit six months before the current study. Therefore, a sampling interval of ≈3 was assumed by dividing 877 by the calculated sample size (877/300 ≈ 3), and eligible women were interviewed for every three intervals until the required sample size was reached.

### 2.5. Data Collection

Data were obtained via direct interviews and a questionnaire. Two junior female doctors were trained to counsel the women and collect the data. Medical and socioeconomic data, including age, parity, residence, educational level, past personal or family history of depression, and previous miscarriage or intrauterine fetal death, were collected. The women’s weight and height were measured using the standard procedures, and their body mass index (BMI) was computed.

An Arabic version of the Edinburgh Postnatal Depression Scale (EPDS) was used to assess postpartum depression. The scale consists of 10 questions about how a woman has been feeling in the past seven days, and each question is answered using a scale of 0 to 3. A score of 12 or more is considered “test positive”. The questionnaire aims to identify cases of postpartum depression and its associated factors among mothers who gave birth in the previous six weeks.

### 2.6. Sample Size Calculation

The sample size of 300 women was computed using the OpenEpi Menu [28]. We assumed that 30.0% of the women would have postpartum depression. This assumption was based on the prevalence of depression (35.6%) that we found in patients with diabetes mellites in the same hospital (Gadarif) in Eastern Sudan [29] and on the prevalence of postpartum depression (12.2% to 33.8%) recently reported in the nearby country of Ethiopia [9]. We then assumed that 25.0% of the women who had postpartum depression would have an education level of the secondary level or above and that 40.0% of the women who had no postpartum depression would have the same level of education. The calculated sample size (300 women) had 80.0% power, a 95.0% confidence interval (CI), and a *p*-value of 0.05.

### 2.7. Statistical Analysis

Version 22.0 of the Statistical Package for the Social Sciences^®^ (SPSS^®^) for Windows (SPSS Inc., New York, NY, USA) was used to analyze the data. Categorized data were expressed as frequencies (%). A Shapiro–Wilk test was used to evaluate the normality of the continuous variables, which were found to be not normally distributed and were expressed as medians (interquartile ranges (IQRs)). Univariate analysis was conducted with postpartum depression as the dependent variable. The independent variables were age, parity, BMI, educational level, occupation, a history of previous miscarriage or intrauterine fetal death, a family history of depression, financial support, medical insurance, whether the pregnancy was planned or not, and whether the gender of the newborn was known before delivery or not (these factors were reported to be associated with postpartum depression). We planned to shift variables with *p* < 0.05 to build up the binary multivariate analysis to rule out confounders; however, only one variable was detected, so the model was not built. Odds ratios (ORs) and 95% CIs were calculated as they were applied. A two-sided *p*-value of <0.05 was considered statistically significant.

## 3. Results

The sociodemographic characteristics of the 300 women in the study are shown in Table 1. The median (IQR) of the age, parity, and BMI was 30.0 (25.0–34.0) years, 2 (1–4), and 28.9 (26.04–31.5) kg/m^2^, respectively. The number of women with education beyond the primary level was 158 (52.7%), and 251 (83.7%) lived in urban areas. Of the participants, 256 (85.3%) were housewives, only 44 (14.7%) were employed, 143 (47.7%) had medical insurance, and 251 (83.7) had urban residences. Twenty-one (7%) of the women had a previous history of depression, and 43 (14.3%) had a family history of depression. The pregnancy was unplanned for 167 (55.7%) of the women, 45 (15%) had a history of intrauterine fetal death, 80 (26.7%) had a history of miscarriage, and 31 (10.3%) had depressive symptoms (EPDS ≥ 12).

A univariate analysis revealed that a past history of depression was the only factor associated with postpartum depression (OR = 3.04, 95% CI = 1.03–8.97). The other investigated factors (age(OR = 0.99, 95% CI = 0.94–1.06), parity (OR = 1.02, 95% CI = 0.85–1.23), BMI (OR = 0.91, 95% CI = 0.83–1.01), educational level (OR = 0.47, 95% CI = 0.20–1.06), occupation (OR = 1.13, 95% CI = 0.41–3.13), history of previous miscarriage (OR = 1.13, 95% CI = 0.41–3.13), or intrauterine fetal death (OR = 1.21, 95% CI = 0.40–3.65), family history of depression (OR = 3.04, 95% CI =1.03–8.97), lack of financial support (OR = 0.33, 95% CI = 0.06–1.72), no medical insurance (OR = 0.97, 95% CI = 0.46–2.04), unplanned pregnancy (OR = 1.29, 95% CI = 0.60–2.77), and the sex of the newborn was known before delivery (OR = 0.82, 95% CI = 0.51–1.31)) were not associated with postpartum depression (Table 2). Therefore, the variables were not shifted to a multivariate analysis.

## 4. Discussion

The current study showed that 10.3% of the women had postpartum depression. This observed prevalence is similar to the prevalence of postpartum depression previously reported in Khartoum, the capital of Sudan [17,22], in Malawi(9.6%) [30] and to the mean global prevalence of 11.0% previously reported in a large multinational study conducted in 138 countries [18]. However, the reported prevalence of postpartum depression in the current study is higher than that in the neighboring countries of Eretria (7.4%) [12], in Morocco (6.9%) [6], In Egypt (3.7% and 33.5%) [8]. The 10.3% prevalence of postpartum depression in this study is lower than that reported in Ethiopia (12.2–33.8%) [9], Kenya (27.1%) [13], Uganda (27.1%) [10], Nigeria (37.8%) [4], Somalia (59.9%) [14], Cameroon (31.8%) [15], Burkina Faso (44.0%) [6], South Africa (39.96%) [2], and Tanzania (12.2%) [11] and lower than the overall reported prevalence of postpartum depression in countries with fewer resources (14.8%) [2]. The great variation in the prevalence of postpartum depression in different populations could be explained by the differences in the sociodemographic characteristics, cultural, socio-economic, and the timing and method of evaluation. Perhaps the actual prevalence of postpartum depression is underestimated, and some communities could consider accessing and diagnosing mental illness as a social stigma [5]. Consequently, the available statistics are limited [31].

The current study showed that women with a past history of depression have 3.04 times more risk of having postpartum depression. This is consistent with a previous study in Cameroon showing that a family history of mental health illness and a previous history of depression was associated with increased odds of postpartum depression [15]. In a meta-analysis conducted in Ethiopia, Tolossa et al. showed that the odds of developing postpartum depression were 4.52 times higher among women who had a previous history of depression [7]. Other sociodemographic variables, such as age, parity, maternal employment, residence, family history of mental illness, medical insurance, a history of abortion or intrauterine fetal death, and BMI, were not associated with postpartum depression in this study. In Ethiopia, maternal education and household socioeconomic status are not associated with postpartum depression [24]. In Somalia, educational level, residence, occupation, and parity are not associated with postpartum depression [14]. However, in Khartoum, Sudan, age is associated with postpartum depression [19], and in Cameroon, gender-based violence, financial stress, and having a male baby are associated with postpartum depression [15]. In Ethiopia, a meta-analysis that included 11 studies and 7582 women showed that unplanned pregnancy, domestic violence, and poor social support were associated with postpartum depression [9]. In Kenya, age, unplanned pregnancy, and a low level of education have been shown to be associated with postpartum depression [13]. In Malawi, live birth outcome, marital status, and lower educational level have been shown to be associated with decreased odds of having postpartum depression [30]. Previous studies have not shown an association between the level of education and postpartum depression [19,32]. However, still, others have shown that having a higher educational level decreases the odds of postpartum depression [16,22,33]. Several previous studies have shown that obese women are at higher risk for postpartum depression [33,34], although our study found no association between obesity and postpartum depression. The non-significant association between these factors and postpartum depression in our study and the other studies could be explained by the differences in study design, sample size, or population characteristics.

## 5. Conclusions

The current study showed that 1 out of 10 participating women had postpartum depression that was associated with a past history of depression. Other factors (age, parity, education, and BMI) that have been reported to be associated with postpartum depression in other African countries were not associated with the condition in our study. Nationally, postpartum depression should be considered an important determinant of maternal and child morbidity, and efficient educational and screening programs should be implemented at postnatal clinics.

## 6. Limitations of the Study

The main limitation of our study is that it is a single-center study, and the results might not be representative of other parts of Sudan. The study is facility-based, so it might not reflect what is happening in the community. Moreover, the cross-sectional design, the self-report instruments, and the potential for selection bias with the systematic random sampling technique must be mentioned. In addition, women seeking postnatal care and perhaps women with postpartum depression or another illness could have been managed somewhere else, perhaps by doctors in a psychiatric department. We did not build a multivariate model due to only one significant variable in the univariate analysis, and this carries a potential bias. Research suggests that inflammation may play a role in the pathophysiology of depression, including postpartum depression [35], and this was not assessed.

## Figures and Tables

**Table 1:** The sociodemographic characteristics of postpartum women (number = 300) in Eastern Sudan, 2022.

Variables			
***The median (interquartile range) of***		**Median**	**Interquartile range**
Maternal age, years		30	25.0–34.0
Parity		2	1–4
Body mass index, kg/m^2^		28.9	26.04–31.5
***Frequency (proportion)***		**Total (N = 300)**	**Percentage (%)**
Financial support	No	292	97.3
	Yes	8	2.7
Medical insurance	No	157	52.3
	Yes	143	47.7
Maternal education status	≥Primary	166	**53.3**
	<Primary	134	**44.7**
Maternal employment status	Employed	44	14.7
	Housewife	256	85.3
Residence	Rural	49	16.3
	Urban	251	83.7
Planned pregnancy	No	167	55.7
	Yes	133	44.3
Did you know the baby’s sex before delivery	No	135	45.0
	Yes	165	55.0
History of miscarriage	No	172	57.3
	Yes	138	42.7
History of intrauterine fetal death	No	255	85.0
	Yes	45	15.0
Past history of depression	No	279	93.0
	Yes	21	7.0
Family history of depression	No	257	85.7
	Yes	43	14.3

**Table 2:** Univariate analysis of factors associated with postpartum depression in women (number = 300) in Eastern Sudan, 2022.

Variables		Women with Postpartum Depression N = 31	Women without Postpartum Depression N = 269	Odds Ratio (95% Confidence Interval)	*p*-Value
		*The median (interquartile range) of*			
Maternal age, years		30.0 (23.0–35.0)	29 (25.0–34.0)	0.99 (0.94–1.06)	0.899
Parity		2 (1–4)	2 (1–4)	1.02 (0.85–1.23)	0.831
Body mass index, kg/m^2^		28.3 (24.3–30.0)	29.0 (26.2–31.6)	0.91 (0.83–1.01)	0.063
	*Frequency (proportion)*				
Financial support	No	29 (93.5)	263 (97.8)	0.33 (0.06–1.72)	0.188
	Yes	2 (6.5)	6 (2.2)	Reference	
Medical insurance	No	16 (51.6)	141 (52.4)	0.97 (0.46–2.04)	0.932
	Yes	15 (48.4)	128 (47.6)	Reference	
Maternal education status	≥Primary	22 (71.0)	144 (53.5)	Reference	0.069
	<Primary	9 (29.0)	125 (46.5)	0.47 (0.20–1.06)	
Maternal employment status	Employed	5 (16.1)	39 (14.5)	1.13 (0.41–3.13)	0.808
	Housewife	26 (83.9)	230 (85.5)	Reference	
Residence	Rural	6 (19.4)	43 (16.0)	0.79 (0.31−2.04)	0.631
	Urban	25 (80.6)	226 (84.0)	Reference	
Planned pregnancy	No	19 (61.3)	148 (55.0)	1.29 (0.60–2.77)	0.507
	Yes	12 (38.7)	121 (45)	Reference	
Did you know the baby’s sex before delivery?	No	13 (41.9)	122 (45.4)	0.82 (0.51–1.31)	0.402
	Yes	18 (58.1)	147 (54.7)	Reference	
History of abortion	No	16 (51.6)	156 (58)	Reference	0.150
	Yes	15 (48.4)	113 (42)	1.33 (0.90–1.96)	
History of intrauterine fetal death	No	27 (87.1)	228 (84.8)	Reference	0.730
	Yes	4 (12.9)	41 (15.2)	1.21 (0.40–3.65)	
Past history of depression	No	26 (83.9)	253 (94.1)	Reference	0.044
	Yes	5 (16.1)	16 (5.9)	3.04 (1.03–8.97)	
Family history of depression	No	23 (74.2)	234 (87)	Reference	0.060
	Yes	8 (25.8)	35 (13)	0.43 (0.18–1.04)	

## Data Availability

The data supporting the current study’s findings are available from the corresponding author upon reasonable request.
